# Supplementary material for: End Sequence Analysis Toolkit (ESAT) expands the extractable information from single-cell RNA-seq data
Source: Genome Res. 2016 Oct;26(10):1397–410. doi: 10.1101/gr.207902.116 (PMC5052061; doi:10.1101/gr.207902.116)
Supplement: Supplemental Material [file supp_gr.207902.116_Supp_Materials.tar.gz › ESAT-esat_v0.1/doc/html/functions_vars_p.html]

ESAT: Class Members - Variables


ESAT


- Main Page
- Related Pages
- Packages
- Classes
- Files

- Class List
- Class Index
- Class Hierarchy
- Class Members

- All
- Functions
- Variables
- Enumerator

- a
- b
- c
- d
- e
- f
- g
- h
- i
- k
- l
- m
- n
- o
- p
- q
- r
- s
- t
- u
- v
- w


All Classes Namespaces Files Functions Variables Enumerator Pages

### - p -

- PAIRED
  : broad.pda.seq.graph.ChromosomeWithBubblesJGraphT.EdgeSourceType
- PAIRED\_END
  : broad.pda.seq.rap.CollectAnnotationEnrichments
  , broad.pda.seq.rap.GenomeScoringProgram
  , broad.pda.seq.rap.rna.TranscriptomeScoringProgram
- PAIRED\_END\_EXTENSION
  : umms.core.writers.PairedEndWriter
- PAIRED\_SUPPORT
  : broad.pda.seq.graph.ChromosomeWithBubblesJGraphT.EdgeSourceType
- PCT\_MASKED\_ALLOWED
  : broad.pda.seq.rap.GenomeCommandLineProgram
- PERMUTATION\_ATTEMPTS
  : umms.core.coordinatesystem.GenomicSpace
  , umms.core.coordinatesystem.TranscriptInGenomicSpace
  , umms.core.coordinatesystem.TranscriptomeSpace
- PERMUTATIONS
  : broad.pda.seq.rap.BuildRatioNullDistribution
  , broad.pda.seq.rap.CollectAnnotationEnrichments
  , broad.pda.seq.rap.ShuffleBAM
  , broad.pda.seq.rap.ShuffleBED
- PERMUTE
  : broad.pda.seq.rap.CollectAnnotationEnrichments
- POINT\_MATH
  : broad.pda.seq.rap.DistanceToBED
- POINT\_PATTERN
  : broad.core.annotation.BasicGenomicAnnotationReader
- POSITIVE
  : umms.core.annotation.Annotation.Strand
- PROTEIN
  : jaligner.Sequence
- pValueCutoffScan
  : broad.pda.seq.protection.TwoSampleScanSkellamPeakCaller
- pValueCutoffSkellam
  : broad.pda.seq.protection.TwoSampleScanSkellamPeakCaller


---

Generated on Fri Aug 15 2014 15:11:17 for ESAT by  

 1.8.7
